# Nutritional Support in the Prevention and Treatment of Pressure Ulcers in Healthy Aging: A Systematic Review of Nursing Interventions in Community Care

**DOI:** 10.3390/geriatrics10010017

**Published:** 2025-01-22

**Authors:** Giovanni Cangelosi, Francesco Sacchini, Federico Biondini, Stefano Mancin, Sara Morales Palomares, Gaetano Ferrara, Gabriele Caggianelli, Marco Sguanci, Fabio Petrelli

**Affiliations:** 1 Unit of Diabetology, Asur Marche—Area Vasta 4 Fermo, 63900 Fermo, Italy; giovanni.cangelosi@virgilio.it; 2 Nursing Department, Polytechnic University of Ancona, 60121 Ancona, Italy; francescosacchini@libero.it; 3 Units of Psychiatry, Ast Fermo, 63900 Fermo, Italy; federico.biondini@sanita.marche.it; 4 IRCCS Humanitas Research Hospital, Via Manzoni 56, 20089 Rozzano, Italy; 5 Department of Pharmacy, Health and Nutritional Sciences (DFSSN), University of Calabria, 87036 Rende, Italy; 6 Nephrology and Dialysis Unit, Ramazzini Hospital, 41012 Carpi, Italy; amaranto1984@libero.it; 7 Azienda Ospedaliera San Giovanni Addolorata, 00184 Rome, Italy; caggianelligabriele@gmail.com; 8 A.O. Polyclinic San Martino Hospital, Largo R. Benzi 10, 16132 Genova, Italy; sguancim@gmail.com; 9 School of Pharmacy, Polo Medicina Sperimentale e Sanità Pubblica “Stefania Scuri”, 62032 Camerino, Italy; fabio.petrelli@unicam.it

**Keywords:** healthy aging, lifestyle medicine, nursing, pressure ulcers, nutritional assessment, community and public health, systematic review

## Abstract

**Introduction:** Pressure ulcers (PUs) represent a significant challenge in chronic care, particularly among the elderly with reduced mobility. They result in substantial socio-healthcare costs and negatively impact patients’ psychological well-being. Malnutrition is a key risk factor, hindering healing and increasing the risk of complications. As such, appropriate nutritional interventions are crucial for managing PUs. However, these interventions are often underestimated in clinical practice, underscoring the need for a more comprehensive approach to elder care and healthy aging. **Objectives:** The primary objective of this study was to identify the best scientific evidence on nutritional interventions implemented by nurses in community settings to prevent complications related to PUs. Additionally, qualitative and quantitative outcomes reported in the included studies were analyzed. **Method:** This systematic review was conducted following PRISMA international guidelines. Searches were performed in PubMed, Scopus, Embase, and CINAHL databases. A predefined search strategy using Boolean operators was employed, and two researchers independently selected papers, with a third researcher resolving any discrepancies. Additional sources and manual reviews were conducted to minimize selection bias. **Results:** Out of 1003 records, 6 studies were included. Findings indicate that nurses play a fundamental role in managing PUs in community settings through specific nutritional intervention assistance processes. These healthcare professionals are pivotal in the prevention, care, and rehabilitation of vulnerable individuals with PUs. **Conclusions:** Nurses are strategic in the management of PUs within community care for frail populations. With nutritional support that plays a key role in both preventing and accelerating the healing of PUs. Policymakers are encouraged to invest in nursing roles to enhance assistance in community contexts, supporting healthy aging and lifestyle medicine approaches.

## 1. Introduction

The global aging population is one of the most significant phenomena of the 21st century. By 2050, the proportion of people aged 60 years or older is expected to rise from 10% in 2022 to 16%, reaching a total of 2.1 billion individuals [United Nations (UN), World Health Organization (WHO)]. Notably, 80% of this demographic will reside in low and middle-income countries, and as of 2020, the number of individuals over 60 years old has already surpassed that of children under five. According WHO data, this trend is accelerating, with the older age group expected to double between 2015 and 2050, constituting 22% of the global population [1,2]. In Italy, the aging trend is particularly pronounced, with the country ranking third in Europe and fifth worldwide for average age (48.4 years) [Central Intelligence Agency (CIA) report] [3]. Projections indicate that by 2050, 35.9% of Italy’s population will be aged 65 or older [Istituto Superiore di Sanità (ISS)] [4]. Advanced age brings a range of complex health challenges, collectively referred to as geriatric syndromes, including hearing problems and chronic diseases [5]. Among these, pressure ulcers (PUs)—localized damage to the skin and/or underlying tissues—are a prevalent issue, particularly among individuals with reduced mobility [6,7]. In Europe, an estimated 1.5–2 million people suffer from chronic wounds, including PUs, accounting for 3% of healthcare expenses [8,9]. Managing PUs poses significant challenges, with profound implications for both patients and healthcare systems. PUs negatively affects patients’ quality of life (QoL), causing pain and psychological distress [10,11], and place a substantial financial burden on healthcare systems [12]. In Italy, over 14 million individuals are aged 75 years or older, with a higher prevalence among women [National Institute of Statistics (ISTAT) report, 2024] [13]. This demographic shift has increased care demands and healthcare costs due to comorbidities and prolonged bed rest, complicating the provision of essential levels of care [14]. Preventing chronic diseases and their complications through targeted educational programs led by nurses is a key strategy for promoting Healthy Aging and Lifestyle Medicine, particularly in community and neighborhood settings [15,16,17]. PUs remains one of the primary nursing-sensitive outcomes [18,19], with prevalence varying across care settings [20]. Despite the significance of the problem, studies focusing on PUs in community settings are limited [21,22,23]. In home care environments, caregivers play a critical role in managing care and require adequate training to enhance lesion management and prevent complications [24,25,26].

A critical factor in PUs development is malnutrition, defined as ESPEN “a state resulting from the deficiency of intake or absorption of nutrients that leads to altered body composition and a reduction in physical and mental function” [27]. Malnutrition is an escalating global issue, driven by demographic aging and the increasing prevalence of age-related diseases [28]. Among elderly, malnutrition represents a multifaceted challenge; nutrition and hydration are not merely physiological needs but also hold substantial social and psychological significance [29]. Protein-energy malnutrition and deficiencies in key micronutrients, particularly vitamins C and E, severely impair skin regeneration, increasing susceptibility to PUs in elderly populations [30]. The prevention and management of PUs require a multidisciplinary and personalized approach that extends beyond medical interventions to include lifestyle and nutritional strategies aimed at supporting overall health and physical functionality in elderly. Healthy Aging strategies underscore the importance of optimal nutrition, regular physical activity, and effective management of chronic conditions, with the goal of promoting longevity and preventing age-related illnesses [31,32]. Within this context, Lifestyle Medicine offers evidence-based interventions that empower individuals to adopt sustainable behavioral changes, fostering a healthier aging process and improving chronic care outcomes [33,34,35].

Nurses play a pivotal role in the comprehensive management of elderly, particularly in identifying and addressing malnutrition risks associated with chronic conditions [36]. They are central to nutritional screening and ongoing monitoring of risk factors for PUs. Nurses adopt a comprehensive approach to evaluate the various physical and emotional factors influencing the health of elderly. This allows them to design and implement specific strategies for nutrition and hydration, targeting individual deficiencies and particular health needs [37]. Additionally, nurses are instrumental in training caregivers, equipping them with the necessary knowledge and skills to support optimal health outcomes. Ultimately, nurses act as key facilitators of preventive and integrated care, collaborating with healthcare professionals who are specifically trained and expert (e.g., dietitians/clinical nutritionists) to reduce the incidence of PUs, mitigate the effects of malnutrition, and enhance the QoL for elderly. Their contributions are vital to promoting overall health and well-being throughout the aging process [38,39].

This study evaluates the impact of nutritional strategies on the prevention and treatment of PUs in elderly receiving community-based nursing care, with a focus on Healthy Aging, functional independence, and clinical outcomes.

## 2. Materials and Methods

### 2.1. Review Design

To ensure the methodological completeness and significance of the included studies, the Systematic Review (SR) involved the preliminary development of a research protocol, followed the PRISMA guidelines (Preferred Reporting Items for Systematic Reviews and Meta-Analyses) (Supplementary File S1 Check list) [40,41], and consulted the Cochrane Handbook for SR [42].

### 2.2. Protocol Registration

The protocol of this SR was registered in the Open Science Framework (OSF) Register database (https://doi.org/10.17605/OSF.IO/SHNJU) (accessed on 16 October 2024) [43].

### 2.3. Study Objectives and Research Question

The main objective of the study was to evaluate the impact of nutrition in the nursing care of PUs within territorial contexts as home care and/or institutional setting (e.g., nursing home, community nursing hospital). Secondly, the quantitative and qualitative results collected were analyzed. The review aimed to answer the following research questions:

*Primary research question:*

What are the most reliable scientific evidence on nutritional interventions implemented by nurses in community settings to prevent complications related to PUs in elderly?

*Secondary research questions:*

Which nutritional strategies are most effective in improving clinical outcomes and preventing PUs in elderly managed in community care settings, with an emphasis on promoting Healthy Aging and functional independence?

What is the relationship between nutritional status and the development or healing of PUs in elderly receiving community-based care, and how do these factors contribute to overall Healthy Aging outcomes?

The research questions were formulated using the PICOS framework [44], consolidated in previous studies [45,46] (Population, Intervention, Comparison, Outcome, Study design), specifically: P, elderly with PUs in community settings in; I, nursing support in nutritional strategies; C, nursing support in nutritional strategies vs. no nursing support in specific nutritional strategy; O, qualitative/quantitative outcomes; and S, primary studies.

### 2.4. Search Strategy

The searches were conducted in PubMed/Medline, Scopus, Embase, and CINAHL databases, with additional exploration of gray literature sources such as Google Scholar. The search strategy utilized keywords including “Diet”, “Community Nurse”, and “Pressure Ulcers”, alongside their variations. Customized search strings were adapted to the specific characteristics of each database. Boolean operators (AND/OR) were employed to refine the search and identify studies aligned with the research objectives. Search limits were applied, focusing on study type, thematic relevance, language, and publication date (Supplementary File S2).

The free version of the citation management software Mendeley (Version 2.120.0) [47] facilitated the compilation of the database and the elimination of duplicates. To minimize selection bias, manual screening of articles was performed, and the bibliographies of included studies were carefully examined for additional relevant records.

### 2.5. Inclusion and Exclusion Criteria

The inclusion criteria comprised primary studies mainly in English, relevant to the study’s objective involving elderly individuals (scientifically referred to as those over sixty-five), without time limits.

Exclusion criteria encompassed all non-primary study types (e.g., editorials, commentaries, reviews, and protocols). Studies involving heterogeneous populations compared to the one studied those written in Chinese were also excluded.

### 2.6. Quality Assessment and Risk of Bias Evaluation

The risk of bias and the methodological quality of the included records were independently assessed by two researchers (F.S. and G.C.) using a double-blind methodology. Any disagreements were resolved by a third impartial researcher (S.M.). For the assessment, the Critical Appraisal Skills Programme (CASP) checklists [48] were employed (Supplementary Files S3 and S4 for excluded studies). This rigorous evaluation ensured that the conclusions drawn are based on high-quality evidence, contributing to reliable findings for the research question.

### 2.7. Data Extraction and Synthesis

The data from the selected records were extracted and reported in a specific data extraction table that included Author(s), Year and Country of Study, Type of Study, Study Cohort, Intervention(s), Bias, and Main Results. These elements were reported as a narrative summary and integrated into Figures and Tables to address the review’s objectives comprehensively.

## 3. Results

**Figure 1:**
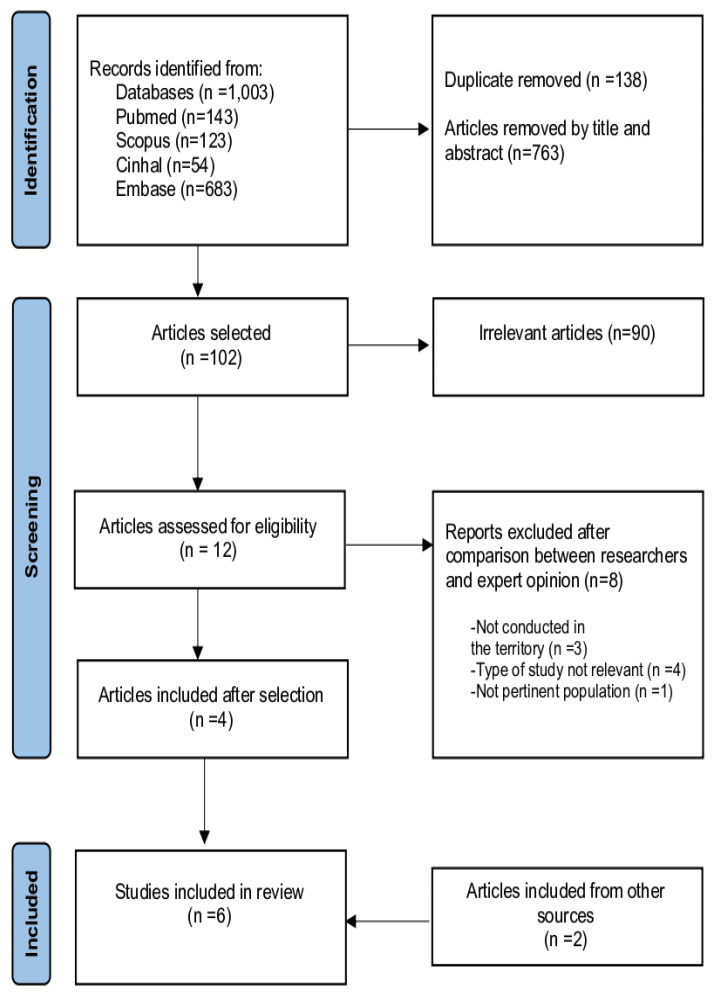
PRISMA flowchart.

A total of 1003 articles were identified through searches in four databases: 143 from PubMed, 123 from Scopus, 54 from CINAHL, and 683 from Embase. After removing 901 articles (138 duplicates and 763 based on title and abstract), 102 articles were selected for further review. Of these, 90 were excluded as irrelevant, and 12 full-text articles were assessed for eligibility. Upon further evaluation by the researchers and expert opinion, 8 articles were excluded, with details provided in Supplementary File S3. Ultimately, 6 primary studies conducted between 1998 and 2019 were included in the review, 2 of which were identified from other sources [49,50,51,52,53,54] (PRISMA flowchart in Figure 1 and Summary of the included studies in Table 1).

### 3.1. Characteristic of the Included Studies

**Figure 2:**
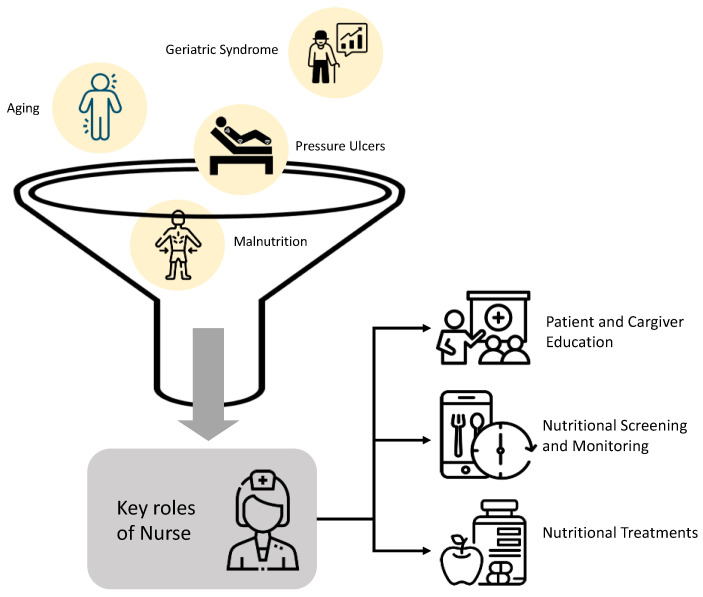
Graphic Representation of the study.

Of the 7 primary studies included [49,50,51,52,53,54], two were conducted in Italy, three in the United States and Canada, one in Belgium, and Luxembourg. Four studies were Randomized Clinical Trials (RCT), one Clinical Trial (CT), and two observational studies. The publication year of the included studies ranges from 2005 to 2019; two studies were published in 2015, one in the 2005, 2008, 2009, and 2019. A total of 1710 subjects were involved: 1202 in the experimental groups and 508 in the control groups. Five studies demonstrated adequate methodological quality and a low risk of bias, while only one study had uncertain methodological quality [46] (Table 2 and Figure 2).

### 3.2. Systematic Results of the Included Studies

#### 3.2.1. Nursing Assessment of Nutritional Status and Risk of PUs

Yap T et al. [49] conducted an observational study in 2019 across seven nursing homes in the metropolitan area of Toronto over a 21-day period. The study data were analyzed to compare differences in nutritional status, food intake, and non-nutritional risk factors for PUs between Asian patients (PA, n = 97) and non-Asian patients (PNA, n = 408) (average age: 85.91; females: 306). Certified Nursing Assistants (CNAs) performed assessments in nutritional assistance and support for care management, while PUs were evaluated using the Braden Scale. Results indicated that PA had a significantly lower average body mass index (BMI) than PNA (21.7 vs. 27.8). Additionally, PA showed a higher percentage of full meal consumption (70.3% vs. 46.2%) and experienced more wet slips (5.13 vs. 3.56) compared to PNA. Supporting these findings, Kennerly S et al. [50] conducted an observational study in 2015 involving 27 patients (average age: ≥65) at moderate or high risk of developing PUs in nursing homes in the United States and Canada. CNAs screened patients using the Braden Scale, including the nutritional risk subscale, over a three-week period. Food intake and weight changes were also monitored. Patients classified in category 1 (very poor) of the nutritional subscale exhibited significantly lower BMIs compared to those in category 4 (excellent) (22.3 vs. 27.8 kg/m^2^) and lower total Braden scores (11.8 vs. 12.9). Meal intake percentages also differed significantly, with patients in category 3 (adequate) consuming 69.8–74.8% of meals, compared to 97.4% in category 4 (excellent).

#### 3.2.2. Nursing Nutritional Interventions and Promotion of PUs Healing

In 2015, Cereda E et al. [51] conducted a multicenter, double-blind RCT in long-term care and home care facilities in Italy, involving 200 patients (average age: 81.4 ± 10.73; females: 137) over 8 weeks. The experimental group (EG, n = 101) received a hypercaloric/protein oral nutritional formula enriched with arginine, zinc, and antioxidants, while the control group (CG, n = 99) was given a standard formula. Trained nurses assessed PUs healing using the Braden Scale. Results showed that the EG had a greater reduction in PUs size (60.9% vs. 45.2% in the CG) and a higher rate of complete healing at 4 weeks (16.9% vs. 9.7%). Further evidences come from a 2009 multicentric RCT by Cereda E et al. [52] conducted in four long-term care facilities in Como, Italy. Twenty-eight patients with stage II or higher PUs were randomized into two groups: the CG (n = 15), which received standard nutritional care, and the EG (n = 13; average age: 81.73 ± 9.59; females: 18), which was given a high-protein supplement enriched with arginine, zinc, and vitamin C. All participants received standard wound care and were assessed by nurses using the Pressure Ulcer Scale for Healing (PUSH) and the Norton Scale for 12 weeks. The EG demonstrated superior outcomes, with a significant reduction in PUSH scores (7.4 ± 3.4 vs. 10.7 ± 3.4 in the CG) and a larger average reduction in PU area after 8 weeks (57% vs. 33%). Notably, dietary adherence was high in both groups (94.3% in the CG and 94.7% in the EG).

#### 3.2.3. Nursing Monitoring and Intensive Management of PUs

A multicenter, open clinical trial conducted by Heyman H et al. [53] in 2008 involved 61 nursing homes across Belgium and Luxembourg. The study recruited 245 patients (average age: 82.2 ± 10.1; females: 59) with stage II-IV PUs to evaluate the effects of oral nutritional supplements (ONS) administered for 9 weeks alongside standard care. PUs measurements were performed by healthcare providers using a millimeter ruler, and an evaluation questionnaire assessed compliance. Patients consumed an average of 2.3 ± 0.56 ONS servings daily, with 35% (n = 86) rating compliance as excellent and 47% (n = 115) as very good. Over the 9-week period, the average PUs area significantly reduced by 53% (from 1580 ± 3743 mm^2^ to 1103 ± 2999 mm^2^). Additionally, 9 out of 10 operators expressed support for continued use of ONS after the intervention. Similarly, Stechmiller J et al. [54] conducted a multicenter RCT in 2005 across nursing homes in Florida, USA, involving 26 patients with one or more PUs. Participants were randomized into an experimental group (EG, n = 14) and a control group (CG, n = 12), with an average age of 78.49 ± 1.76 (females: 1). Nurses administered nutritional supplements and evaluated nutritional status using the Mini Nutritional Assessment (MNA) over 4 weeks. The EG received 8.5 g of arginine supplements, while the CG received isonitrogenous supplements. Although serum arginine levels in the EG increased significantly at 4 weeks (128.7 ± 12.3 vs. 113.2 ± 10.0 in the CG), they decreased at 10 weeks (95.4 ± 8.2). Serum nitric oxide levels in the EG remained stable, with no significant changes observed at 4 weeks (65.8 ± 16.9) or 10 weeks (51.7 ± 9.6) compared to baseline (66.9 ± 19.8).

## 4. Discussion

PUs affect the health of over 7 million people worldwide [55], significantly impairing the QoL of those affected [56]. These lesions not only compromise the physical well-being of elderly but also profoundly impact their emotional and social health, leading to chronic pain, reduced mobility, and increased dependency. This loss of independence, a critical determinant of Healthy Aging, underscores the importance of effective strategies for PUs prevention and management. Furthermore, the economic burden of PUs extends to caregivers, families, and healthcare systems, highlighting the need for resource-efficient approaches [57].

In the context of Healthy Aging, preventing and managing PUs is essential for maintaining autonomy, dignity, and functional capacity in elderly. A multifaceted approach, grounded in Lifestyle Medicine principles such as proper nutrition, hydration, and physical activity, can mitigate the risk of PUs and enhance overall well-being. Consequently, addressing PUs is a vital component of strategies aimed at enhancing healthy aging and supporting individuals in maintaining independence and QoL [58,59].

Malnutrition, a primary risk factor for PUs [60], has shown to be addressable through targeted nutritional interventions, offering significant potential for improvement in both prevention and treatment outcomes [61]. Nurses, as the healthcare professionals with the most direct and frequent patient interactions [62], are uniquely positioned to play a pivotal role in providing nutritional support, particularly in community-based care environments [63]. While much research has focused on PUs acquired in hospital settings, relatively little attention has been given to their occurrence in community, home, or nursing home settings [64], despite the prevalence of these lesions in such environments [65].

This study underscores the essential role of nutrition in the prevention and nursing management of PUs within territorial care settings. The findings highlight the importance of nursing interventions in assessing, managing, and monitoring nutritional status to enhance clinical outcomes and optimize healthcare resource allocation. Identifying malnutrition risk through valid and reliable tools is a critical first step in initiating timely, personalized nutritional interventions, particularly in the context of Healthy Aging.

The use of tools such as the Braden Scale, which includes a nutritional subscale, has proven effective in identifying patients at nutritional risk and determining dietary adequacy in elderly populations [66]. Numerous studies have confirmed its widespread application as a reliable method for detecting at-risk individuals, demonstrating a strong balance of sensitivity and specificity [67,68,69]. Other tools, such as the Mini Nutritional Assessment (MNA) and the Malnutrition Universal Screening Tool (MUST), also play vital roles in nutritional screening, particularly in daily clinical practice. While the MUST is quick and easy to use, assessing key indicators such as BMI and weight loss, it is essential to consider that current international BMI thresholds, based on Western populations, may not be suitable for all ethnic groups, such as Asians, who have distinct physiological and dietary needs [70,71].

In territorial care settings, implementing tailored nutritional plans becomes crucial for preventing and treating PUs. Adequate nutrition is a critical factor in promoting healing and improving the QoL for elderly [72,73,74,75,76]. This study demonstrated the effectiveness of specific nutritional interventions, such as arginine-enriched supplements, over standard care, reinforcing the value of targeted nutritional support in improving clinical outcomes [47,48,49,50,51,52]. While research highlights the positive impact of nutritional supplementation on PU healing, it also emphasizes the need to carefully manage energy supplement administration to avoid potential adverse effects [77,78].

Nurses play a central role in supporting Healthy Aging by continuously monitoring skin conditions, evaluating nutritional status, and ensuring adherence to tailored nutritional plans. In community settings, where healthcare personnel may have less frequent contact with patients, active participation from both patients and caregivers is crucial. This involvement can be enhanced through educational materials and practical guidance on nutrition management, promoting continuity of care and optimizing patient outcomes [79].

Adopting a multidisciplinary approach is fundamental to the prevention and management of PUs. The collaborative integration of nurses, dietitians, and physicians ensures a comprehensive strategy for nutritional support, improving clinical outcomes and alleviating the burden of PUs on healthcare systems [55]. The expertise and dedication of healthcare professionals to promoting optimal nutritional practices are essential for effectively addressing malnutrition in the elderly. This approach results in improved health outcomes, reduced PUs incidence and healing times, and more efficient use of healthcare resources. By focusing on Healthy Aging and emphasizing the critical role of nutrition, these interventions contribute to long-term well-being and foster a healthier, more independent aging process [80,81,82].

### 4.1. Future Research Perspectives

Future research on the prevention and management of PUs should prioritize areas that enhance the role of nurses within the framework of Healthy Aging. A key focus should be the impact of nursing interventions in community care settings, particularly among older populations, which remain under-researched in terms of effectiveness and long-term outcomes. Evaluating educational programs and support strategies for patients and caregivers is essential, as these interventions empower individuals to better manage their health in community contexts. Additionally, integrating multidisciplinary care models—incorporating nurses, physicians, and dietitians—is critical for promoting holistic, age-appropriate care that improves the QoL for elderly and fosters better clinical outcomes. Digital health technologies, such as wearable devices and telemedicine, offer promising opportunities for continuous nutritional and clinical monitoring, particularly for older adults in remote areas. These tools can optimize nursing time in chronic disease management and social care environments [83,84,85]. In Lifestyle Medicine view, it would be appropriate to consider integrative interventions such as physical exercise or passive stimulation provided by experts as a supportive measure for the management or prevention of PUs in the home care setting and chronic care in general [86,87,88,89,90,91]. Furthermore, personalized interventions, including tailored nutritional plans that address the clinical conditions, preferences, and cultural backgrounds of aging individuals, are essential. Such approaches ensure that interventions are both effective and sensitive to the diverse needs of aging populations. Finally, examining the economic impact of nursing practices in the prevention and management of PUs is vital to determine how these strategies can reduce healthcare costs, particularly in community-based settings, while improving the efficiency of healthcare resource utilization. These research directions aim to refine nursing practices within the context of Healthy Aging, supporting sustainable, individualized approaches to care that address the complexities and challenges of aging, chronic conditions, and PUs prevention.

### 4.2. Limitations

The research presents some limitations. Several included studies had small sample sizes, which restricts the generalizability of the findings and complicates the feasibility of conducting a meta-analysis. The presence of comorbidities among participants may have influenced the outcomes, making it challenging to establish a direct correlation between nutritional interventions and their effects. Additionally, one of the studies was neither randomized nor placebo-controlled, thereby increasing the risk of bias. The short duration of the studies also limits the ability to evaluate long-term effects. Lastly, the heterogeneity of the studied samples further hampers the generalization of the results.

## 5. Conclusions

The prevention and management of PUs represent a significant challenge, particularly in community care settings where the aging population is rapidly increasing. This review underscores the vital role of nurses in implementing personalized nutritional interventions as part of a multidisciplinary approach. Malnutrition, a major risk factor for PUs, can be effectively mitigated through appropriate nursing-led strategies, which improve clinical outcomes, accelerate healing, and enhance patients’ QoL.

The prevention and management of PUs represent a significant challenge, particularly in community care settings where the aging population is rapidly increasing. This review highlights the promising role of nurses in implementing personalized nutritional interventions as part of a multidisciplinary approach. Malnutrition, a recognized risk factor for PUs, can be addressed through targeted nursing strategies, which may contribute to improving clinical outcomes, accelerating healing, and potentially enhancing patients’ QoL.

The strategic role of the nurse in PUs management is crucial both in terms of the direct management of the proposed interventions related to nutrition, as well as for the development of specific individualized care plans that focus on the patient and their community.

To maximize the impact of these interventions, continuous training for nursing staff should emphasize patient and caregiver education. This empowerment enables better self-care and reduces the incidence and severity of PUs. Moreover, integrating nutrition-focused strategies into Lifestyle Medicine and Healthy Aging frameworks fosters a proactive, preventive approach to chronic care.

Future research should confirm these findings and explore innovative educational and digital strategies to promote self-management among patients and caregivers. By focusing on sustainable and individualized care models, the healthcare community can address the complexities of aging and support healthier, more independent lives for elderly.

## Figures and Tables

**Table 1:** Summary of the Included Studies.

First Author/ Period/Location	Type of Study	Cohort	Main Intervention and Nusing Tools Adopted	Main Biases	Main Results
Yap T et al. [49]/ 2019/Toronto, Canada	Observational	EG1: 97 (PA), EG2: 408 (PNA); average age: 85.91; females: 306	Comparison of nutritional status, food intake, and non-nutritional risk factors for the development of PUs; tools: Braden Scale	Small sample size; Lack of specificity for Asian subgroups; Missing data on fecal content	Average BMI EG1: 21.7, EG2: 27.8; Complete daily meal consumption EG1: 70.3%, EG2: 46.2%; Wet slips EG1: 5.13, EG2: 3.56
Kennerly S et al. [50]/ 2015/United States and Canada	Observational	27; average age: ≥65	Screening PUs and nutritional risk, monitoring food intake and weight change; tools: Braden Scale	Short study; Limited generalizability	Differences in Braden nutritional subscale: BMI category 1: 22.3 vs. category 4: 27.8; Total Braden category 1: 11.8 vs. category 4: 12.9; Meal intake category 3: 69.8–74.8% vs. category 4: 97.4%
Cereda E et al. [51]/2015/Italy	RCT, multicenter, blind	EG: 101, CG: 99; average age: 81.4 ± 10.73; females: 137	Enriched formula (EG) vs. standard (CG); tools: Braden Scale	Exclusion of patients with normal nutritional status; inclusion of patients able to drink ONS	Ulcer reduction EG: 60.9%, CG: 45.2%; Complete healing at 4 weeks EG: 16.9%, CG: 9.7%
Cereda E et al. [52]/ 2009/Como, Italy	Multicenter RCT	EG: 13, CG: 15; average age: 81.73 ± 9.59; females: 18	Protein supplement, arginine, zinc, vitamin C (EG) vs. standard (CG); tools: PUSH, Norton Scale	Reduced sample; Absence of CG with only proteins; Patients with a recent history of ulcers	PUSH score at 12 weeks EG: 7.4 ± 3.4, CG: 10.7 ± 3.4; Ulcer area reduction EG: 57%, CG: 33%; high dietary adherence in both groups (CG: 94.3%, EG: 94.7%).
Heyman H et al. [53]/ 2008/Belgium and Luxembourg	Clinical trial, multicentric, open	245; average age: 82.2 ± 10.1; females: 59	ONS added to standard care; tools: ruler, questionnaire	Non-randomized study; Lack of inter-evaluator reliability between centers	Daily ONS: 2.3 ± 0.56; Self-reported compliance 35% excellent, 47% very good; Ulcer area reduction after 9 weeks: 53%; 9/10 operators in favor of reusing ONS
Stechmiller J et al. [54]/ 2005/Florida, USA	Multicenter RCT	EG: 14, CG: 12; average age: 78.49 ± 1.76; females: 1	8.5 g arginine supplement (EG) and isonitrogenous supplement (CG); tools: MNA	Small sample size; Limited generalizability; Short study	Serum arginine EG: 128.7 ± 12.3 at 4 weeks, 95.4 ± 8.2 at 10 weeks; no significant change in serum nitric oxide levels at 4 (65.8 ± 16.9) or 10 weeks (51.7 ± 9.6) compared to baseline (66.9 ± 19.8).

**Legend.** EG: experimental group; CG: control group; ONS: Oral Nutritional Supplements; PUs: Pressure Ulcers; PA: Asian Patients; PNA: Non-Asian Patients; BMI: Body Mass Index; PUSH: Pressure Ulcer Scale for Healing; MNA: Mini Nutritional Assessment; RCT: Randomized Controlled Trial; Braden Scale: Tool to assess risk of developing pressure ulcers; Norton Scale: Tool to evaluate the risk of pressure ulcer development; Kuntzmann Scale: Tool for measuring pressure ulcer severity.

**Table 2:** Characteristics of the Included Studies.

Characteristic	Frequency (n = 6)	Percentage
**Publication year**		
2019	1	16.60%
2015	2	33.20%
2009	1	16.60%
2008	1	16.60%
2005	1	16.60%
**Cohort**		
EG	905	86.70%
CG	126	13.30%
**Quality of studies**		
Positive	5	83.40%
Negative	0	0%
Unknowns	1	16.60%

Legend. CG: control group; EG: experimental group.

## Data Availability

Data supporting this research are available in this manuscript and Supplementary Materials.

## Supplementary Materials

The following supporting information can be downloaded at: https://www.mdpi.com/article/10.3390/geriatrics10010017/s1, File S1: Preferred Reporting Items for Systematic Reviews and Meta-Analyses Check-list; Search Strategy File S2; CASP Checklist File S3; Excluded Articles File S4.
