# Supplementary material for: Nutritional Support in the Prevention and Treatment of Pressure Ulcers in Healthy Aging: A Systematic Review of Nursing Interventions in Community Care
Source: Geriatrics (Basel). 2025 Jan 22;10(1):17. doi: 10.3390/geriatrics10010017 (PMC11855182; doi:10.3390/geriatrics10010017)
Supplement: Supplementary file 1 [file geriatrics-10-00017-s001.zip › geriatrics-3412341-supplementary/Supplemenatry Files/Supplementary File S2.pdf]

Supplementary File S2

(Search strategy update November 15, 2024)

Pubmed/Medline Strategy

((((((("Diet"[Mesh] OR "Diet, Mediterranean"[Mesh] OR "Diet Therapy"[Mesh]) OR ("Diet, High-Protein"[Mesh] OR "Diet, Healthy"[Mesh] OR "Diet, Food, and Nutrition"[Mesh])) OR ("Nutritional Sciences"[Mesh] OR "Nutrition Therapy"[Mesh])) OR "Food"[Mesh]) OR ("Food, Fortified"[Mesh] OR "Food, Formulated"[Mesh])) OR "Dietary Supplements"[Mesh]) OR ("diet therapy" [Subheading] OR "Functional Food"[Mesh] OR "Probiotics"[Mesh] OR "Fish Oils"[Mesh])) AND (((((((("Nurses"[Mesh] OR "Family Nurse Practitioners"[Mesh] OR "Nurse Practitioners"[Mesh] OR "Nurse Clinicians"[Mesh] OR "Nurse Specialists"[Mesh] OR "Nurses, Community Health"[Mesh]) OR "Evidence-Based Nursing"[Mesh]) OR "Advanced Practice Nursing"[Mesh]) OR "Family Support"[Mesh]) OR ("Nursing"[Mesh] OR "Home Health Nursing"[Mesh] OR "Family Nursing"[Mesh] OR "Rehabilitation Nursing"[Mesh])) OR ("Holistic Nursing"[Mesh] OR "Nursing Evaluation Research"[Mesh] OR "Clinical Nursing Research"[Mesh] OR "Nursing Research"[Mesh] OR "Skilled Nursing Facilities"[Mesh])) OR "Public Health Nursing"[Mesh]) OR ("Nursing Homes"[Mesh] OR "Nursing Care"[Mesh] OR "Home Nursing"[Mesh] OR "Geriatric Nursing"[Mesh])) OR "Community Health Nursing"[Mesh])) AND ((((((((((((("Pressure Ulcer"[Mesh]) OR (pressure injury)) OR (pressure injuries)) OR (pressure injury prevention)) OR (pressure wound)) OR (pressure wound therapy)) OR (pressure sores)) OR (pressure sore prevention)) OR (pressure sores nursing)) OR (pressure sore)) OR (pressure ulceration)) OR (pressure ulcer treatment)) OR (pressure ulcer prevention)) OR (pressure ulcer prevention nursing))

Filters: no filter

Total results: 143

Scopus Strategy

((TITLE-ABS-KEY ("Pressure Ulcer") OR TITLE-ABS-KEY ("Pressure Injury") OR TITLE-ABS-KEY ("Pressure Injuries") OR TITLE-ABS-KEY ("Pressure Injury Prevention") OR TITLE-ABS-KEY ("Pressure Wound") OR TITLE-ABS-KEY ("Pressure Wound Therapy") OR TITLE-ABS-KEY ("Pressure Sores") OR TITLE-ABS-KEY ("Pressure Sore Prevention") OR TITLE-ABS-KEY ("Pressure Sores Nursing") OR TITLE-ABS-KEY ("Pressure Sore") OR TITLE-ABS-KEY ("Pressure Ulceration") OR TITLE-ABS-KEY ("Pressure Ulcer Treatment") OR TITLE-ABS-KEY ("Pressure Ulcer Prevention") OR TITLE-ABS-KEY ("Pressure Ulcer Prevention Nursing"))) AND ((TITLE-ABS-KEY ("Nurses") OR TITLE-ABS-KEY ("Family Nurse Practitioners") OR TITLE-ABS-KEY ("Nurse Practitioners") OR TITLE-ABS-KEY ("Nurse Clinicians") OR TITLE-ABS-KEY ("Nurse Specialists") OR TITLE-ABS-KEY ("Nurse Community Health") OR TITLE-ABS-KEY ("Evidence Based Nursing") OR TITLE-ABS-KEY ("Advanced Practice Nursing") OR TITLE-ABS-KEY ("Family Support") OR TITLE-ABS-KEY ("Nursing") OR TITLE-ABS-KEY ("Home Health Nursing") OR TITLE-ABS-KEY ("Family Nursing") OR TITLE-ABS-KEY ("Rehabilitation Nursing") OR TITLE-ABS-KEY ("Holistic Nursing") OR TITLE-ABS-KEY ("Nursing Evaluation Research") OR TITLE-ABS-KEY ("Clinical Nursing Research") OR TITLE-ABS-KEY ("Nursing Research") OR TITLE-ABS-KEY ("Skilled Nursing Facilities") OR TITLE-ABS-KEY ("Public Health Nursing") OR TITLE-ABS-KEY ("Nursing Homes") OR TITLE-ABS-KEY ("Nursing Care") OR TITLE-ABS-KEY ("Home Nursing") OR TITLE-ABS-KEY ("Geriatric Nursing") OR TITLE-ABS-KEY ("Community Health Nursing"))) AND ((TITLE-ABS-KEY ("Diet") OR TITLE-ABS-KEY ("Mediterranean Diet") OR TITLE-ABS-KEY ("Diet Therapy") OR TITLE-ABS-KEY ("Diet High Protein") OR TITLE-ABS-KEY ("Diet Healthy") OR TITLE-ABS-KEY ("Diet, Food and Nutrition") OR TITLE-ABS-KEY ("Nutritional Sciences") OR TITLE-ABS-KEY ("Nutrition Therapy") OR TITLE-ABS-KEY ("Food") OR TITLE-ABS-KEY ("Food, Fortified") OR TITLE-ABS-KEY ("Food Formulated") OR TITLE-ABS-KEY ("Dietary Supplements") OR TITLE-ABS-KEY ("Functional Food") OR TITLE-ABS-KEY ("Probiotics") OR TITLE-ABS-KEY ("Fish Oils")))

Filters:

- Subject area: Nursing, Medicine, Health Professions
- Document type: Article
- Language: English, Spanish, French

Total results: 123

Cinhal Strategy

(diet mediterranean OR diet therapy OR diet high-protein OR diet, healthy OR (diet, food, and nutrition) OR nutritional science OR nutrition therapy OR micronutrient supplementation OR food fortified with micronutrients OR food formulation OR dietary supplements OR functional food) AND (nurse OR family nurse practitioner OR nurse practitioner OR nurse clinicians OR nurse specialist OR nursing education OR home health nursing OR family nursing OR rehabilitation nursing OR nursing home OR nursing care OR home nursing) AND (pressure ulcer OR pressure injury OR pressure injury prevention OR pressure wound OR pressure wound therapy OR pressure sore OR pressure sore prevention OR pressure sores nursing OR pressure ulcer treatment OR pressure ulcer prevention OR pressure ulcer prevention nursing OR pressure injuries)

Filters:

- Publication date: from 2000 to 2024
- Language: English, Spanish

Total results: 54

Embase Strategy

('diet'/exp OR 'diet' OR 'mediterranean diet'/exp OR 'mediterranean diet' OR 'high-protein diet'/exp OR 'high-protein diet' OR 'healthy diet'/exp OR 'healthy diet' OR 'food and nutrition' OR 'nutritional science'/exp OR 'nutritional science' OR 'nutrition therapy'/exp OR 'nutrition therapy' OR 'food'/exp OR 'food' OR 'fortified food'/exp OR 'fortified food' OR 'formulated food' OR 'dietary supplements'/exp OR 'dietary supplements' OR 'diet therapy'/exp OR 'diet therapy' OR 'functional food'/exp OR 'functional food' OR 'probiotics'/exp OR 'probiotics' OR 'fish oils'/exp OR 'fish oils') AND ('nurses'/exp OR 'nurses' OR 'family nurse practitioners'/exp OR 'family nurse practitioners' OR 'nurse practitioners'/exp OR 'nurse practitioners' OR 'nurse clinicians'/exp OR 'nurse clinicians' OR 'nurse specialists'/exp OR 'nurse specialists' OR 'community health nurses'/exp OR 'community health nurses' OR 'evidence-based nursing'/exp OR 'evidence-based nursing' OR 'advanced practice nursing'/exp OR 'advanced practice nursing' OR 'family support'/exp OR 'family support' OR 'nursing'/exp OR 'nursing' OR 'home health nursing'/exp OR 'home health nursing' OR 'family nursing'/exp OR 'family nursing' OR 'rehabilitation nursing'/exp OR 'rehabilitation nursing' OR 'holistic nursing'/exp OR 'holistic nursing' OR 'nursing evaluation research'/exp OR 'nursing evaluation research' OR 'clinical nursing research'/exp OR 'clinical nursing research' OR 'nursing research'/exp OR 'nursing research' OR 'skilled nursing facilities'/exp OR 'skilled nursing facilities' OR 'public health nursing'/exp OR 'public health nursing' OR 'nursing homes'/exp OR 'nursing homes' OR 'nursing care'/exp OR 'nursing care' OR 'home nursing'/exp OR 'home nursing' OR 'geriatric nursing'/exp OR 'geriatric nursing' OR 'community health nursing'/exp OR 'community health nursing') AND ('pressure ulcer'/exp OR 'pressure ulcer' OR 'pressure injury'/exp OR 'pressure injury' OR 'pressure injuries' OR 'pressure injury prevention' OR 'pressure wound' OR 'pressure wound therapy' OR 'pressure sores' OR 'pressure sore prevention' OR 'pressure sores nursing' OR 'pressure sore'/exp OR 'pressure sore' OR 'pressure ulceration' OR 'pressure ulcer treatment' OR 'pressure ulcer prevention' OR 'pressure ulcer prevention nursing')

Total results: 683
