# Supplementary material for: Nutritional Support in the Prevention and Treatment of Pressure Ulcers in Healthy Aging: A Systematic Review of Nursing Interventions in Community Care
Source: Geriatrics (Basel). 2025 Jan 22;10(1):17. doi: 10.3390/geriatrics10010017 (PMC11855182; doi:10.3390/geriatrics10010017)
Supplement: Supplementary file 1 [file geriatrics-10-00017-s001.zip › geriatrics-3412341-supplementary/Supplemenatry Files/Supplementary File S3.pdf]

Supplementary File S3

Casp Checklist: For Randomised Controlled Trials (RCTs)

Reference: Cereda E et al. / 2015				
**Section A Is the basic study design valid for a randomised controlled trial?**				
**N**	**Item**	**Yes**	**Can't Tell**	**No**
**1**	Did the study address a clearly formulated research question?	**X**		
**2**	Was the assignment of participants to interventions randomised?	**X**		
**3**	Were all participants who entered the study accounted for at its conclusion?	**X**		
**Section B Was the study methodologically sound?**				
**N**	**Item**	**Yes**	**Can't Tell**	**No**
**4a**	Were the participants 'blind' to intervention they were given?	**X**		
**4b**	Were the investigators 'blind' to the intervention they were giving to participants?	**X**		
**4c**	Were the people assessing/analysing outcome/s 'blinded'?			**X**
**5**	Were the study groups similar at the start of the randomised controlled trial?	**X**		
**6**	Apart from the experimental intervention, did each study group receive the same level of care (that is, were they treated equally)?	**X**		
**Section C: What are the results?**				
**N**	**Item**	**Yes**	**Can't Tell**	**No**
7	Were the effects of intervention reported comprehensively?	**X**		
**8**	Was the precision of the estimate of the intervention or treatment effect reported?	**X**		
**9**	Do the benefits of the experimental intervention outweigh the harms and costs?			**X**
**Section D: Will the results help locally?**				
**N**	**Item**	**Yes**	**Can't Tell**	**No**
**10**	Can the results be applied to your local population/in your context?	**X**		
**11**	Would the experimental intervention provide greater value to the people in your care than any of the existing interventions?	**X**		
**APPRAISAL SUMMARY**				
**Positive/Methodologically sound**	**Negative/Relatively poor methodology**	**Unknowns**		
**X**				

Reference: Heyman H et al. / 2008				
**Section A Is the basic study design valid for a randomised controlled trial?**				
**N**	**Item**	**Yes**	**Can't Tell**	**No**
**1**	Did the study address a clearly formulated research question?	**X**		
**2**	Was the assignment of participants to interventions randomised?			**X**
**3**	Were all participants who entered the study accounted for at its conclusion?	**X**		
**Section B Was the study methodologically sound?**				
**N**	**Item**	**Yes**	**Can't Tell**	**No**

**4a**	Were the participants 'blind' to intervention they were given?			**X**
**4b**	Were the investigators 'blind' to the intervention they were giving to participants?			**X**
**4c**	Were the people assessing/analysing outcome/s 'blinded'?			**X**
**5**	Were the study groups similar at the start of the randomised controlled trial?		**X**	
**6**	Apart from the experimental intervention, did each study group receive the same level of care (that is, were they treated equally)?		**X**	
**Section C: What are the results?**				
**N**	**Item**	**Yes**	**Can't Tell**	**No**
**7**	Were the effects of intervention reported comprehensively?	**X**		
**8**	Was the precision of the estimate of the intervention or treatment effect reported?			**X**
**9**	Do the benefits of the experimental intervention outweigh the harms and costs?		**X**	
**Section D: Will the results help locally?**				
**N**	**Item**	**Yes**	**Can't Tell**	**No**
**10**	Can the results be applied to your local population/in your context?	**X**		
**11**	Would the experimental intervention provide greater value to the people in your care than any of the existing interventions?	**X**		
**APPRAISAL SUMMARY**				
**Positive/Methodologically sound**	**Negative/Relatively poor methodology**	**Unknowns**		
		**X**		

**Reference: Cereda E et al. / 2009**				
**Section A Is the basic study design valid for a randomised controlled trial?**				
**N**	**Item**	**Yes**	**Can't Tell**	**No**
**1**	Did the study address a clearly formulated research question?	**X**		
**2**	Was the assignment of participants to interventions randomised?	**X**		
**3**	Were all participants who entered the study accounted for at its conclusion?	**X**		
**Section B Was the study methodologically sound?**				
**N**	**Item**	**Yes**	**Can't Tell**	**No**
**4a**	Were the participants 'blind' to intervention they were given?	**X**		
**4b**	Were the investigators 'blind' to the intervention they were giving to participants?	**X**		
**4c**	Were the people assessing/analysing outcome/s 'blinded'?		**X**	
**5**	Were the study groups similar at the start of the randomised controlled trial?	**X**		
**6**	Apart from the experimental intervention, did each study group receive the same level of care (that is, were they treated equally)?	**X**		
**Section C: What are the results?**				
**N**	**Item**	**Yes**	**Can't Tell**	**No**
**7**	Were the effects of intervention reported comprehensively?	**X**		
**8**	Was the precision of the estimate of the intervention or treatment effect reported?			**X**
**9**	Do the benefits of the experimental intervention outweigh the harms and costs?		**X**	

Section D: Will the results help locally?				
**N**	**Item**	**Yes**	**Can't Tell**	**No**
**10**	Can the results be applied to your local population/in your context?	**X**		
**11**	Would the experimental intervention provide greater value to the people in your care than any of the existing interventions?	**X**		

APPRAISAL SUMMARY		
**Positive/Methodologically sound**	**Negative/Relatively poor methodology**	**Unknowns**
**X**		

Reference: Stechmiller J et al. / 2005

Section A Is the basic study design valid for a randomised controlled trial?				
**N**	**Item**	**Yes**	**Can't Tell**	**No**
**1**	Did the study address a clearly formulated research question?	**X**		
**2**	Was the assignment of participants to interventions randomised?	**X**		
**3**	Were all participants who entered the study accounted for at its conclusion?	**X**		
**Section B Was the study methodologically sound?**				
**N**	**Item**	**Yes**	**Can't Tell**	**No**
**4a**	Were the participants 'blind' to intervention they were given?	**X**		
**4b**	Were the investigators 'blind' to the intervention they were giving to participants?	**X**		
**4c**	Were the people assessing/analysing outcome/s 'blinded'?	**X**		
**5**	Were the study groups similar at the start of the randomised controlled trial?	**X**		
**6**	Apart from the experimental intervention, did each study group receive the same level of care (that is, were they treated equally)?	**X**		
**Section C: What are the results?**				
**N**	**Item**	**Yes**	**Can't Tell**	**No**
**7**	Were the effects of intervention reported comprehensively?	**X**		
**8**	Was the precision of the estimate of the intervention or treatment effect reported?			**X**
**9**	Do the benefits of the experimental intervention outweigh the harms and costs?		**X**	
**Section D: Will the results help locally?**				
**N**	**Item**	**Yes**	**Can't Tell**	**No**
**10**	Can the results be applied to your local population/in your context?	**X**		
**11**	Would the experimental intervention provide greater value to the people in your care than any of the existing interventions?	**X**		

APPRAISAL SUMMARY		
**Positive/Methodologically sound**	**Negative/Relatively poor methodology**	**Unknowns**
**X**		

Casp Checklist: For Cohort Studies

**Reference: Yap T et al. / 2019**				
**Section A: Are the result valid?**				
**N**	**Item**	**Yes**	**Can't Tell**	**No**
**1**	Did the study address a clearly focused issue?	**X**		
**2**	Was the cohort recruited in an acceptable way?	**X**		
**3**	Was the exposure accurately measured to minimise bias?	**X**		
**4**	Was the outcome accurately measured to minimise bias?	**X**		
**5a**	Have the authors identified all important confounding factors?	**X**		
**5b**	Have they taken account of the confounding factors in the design and/or analysis?	**X**		
**6a**	Was the follow up of subjects complete enough?	**X**		
**6b**	Was the follow up of subjects long enough?	**X**		
**Section B: What are the results?**				
**N**	**Item**	**Yes**	**Can't Tell**	**No**
**7**	What are the results of this study?	**X**		
**8**	How precise are the results?	**X**		
**9**	Do you believe the results?	**X**		
**Section C: Will the results help locally?**				
**N**	**Item**	**Yes**	**Can't Tell**	**No**
**10**	Can the results be applied to the local population?	**X**		
**11**	Do the results of this study fit with other available evidence?	**X**		
**12**	What are the implications of this study for practice?	**X**		
**APPRAISAL SUMMARY**				
**Positive/Methodologically sound**	**Positive/Methodologically sound**			**Unknowns**
**X**				

**Reference: Kennerly S et al. / 2015**				
**Section A: Are the result valid?**				
**N**	**Item**	**Yes**	**Can't Tell**	**No**
**1**	Did the study address a clearly focused issue?	**X**		
**2**	Was the cohort recruited in an acceptable way?	**X**		
**3**	Was the exposure accurately measured to minimise bias?	**X**		
**4**	Was the outcome accurately measured to minimise bias?	**X**		
**5a**	Have the authors identified all important confounding factors?	**X**		
**5b**	Have they taken account of the confounding factors in the design and/or analysis?	**X**		
**6a**	Was the follow up of subjects complete enough?	**X**		
**6b**	Was the follow up of subjects long enough?	**X**		
**Section B: What are the results?**				
**N**	**Item**	**Yes**	**Can't Tell**	**No**
**7**	What are the results of this study?	**X**		

**8**	How precise are the results?	**X**		
**9**	Do you believe the results?	**X**		
**Section C: Will the results help locally?**				
N	**Item**	**Yes**	**Can't Tell**	**No**
**10**	Can the results be applied to the local population?	**X**		
**11**	Do the results of this study fit with other available evidence?	**X**		
**12**	What are the implications of this study for practice?	**X**		
**APPRAISAL SUMMARY**				
**Positive/Methodologically sound**	**Positive/Methodologically sound**			**Unknowns**
**X**				
