# Supplementary material for: Nutritional Support in the Prevention and Treatment of Pressure Ulcers in Healthy Aging: A Systematic Review of Nursing Interventions in Community Care
Source: Geriatrics (Basel). 2025 Jan 22;10(1):17. doi: 10.3390/geriatrics10010017 (PMC11855182; doi:10.3390/geriatrics10010017)
Supplement: Supplementary file 1 [file geriatrics-10-00017-s001.zip › geriatrics-3412341-supplementary/Supplemenatry Files/Supplementary File S4.pdf]

Supplementary File S4 Excluded Articles

Articles excluded after researcher comparison and expert opinion (N=8)

- **Not conducted in a territorial care setting (n=3)**

- Woo HY, Oh SY, Lim L, Im H, Lee H, Ryu HG. Efficacy of nutritional support protocol for patients with pressure ulcer: comparison of before and after the protocol. Nutrition. 2022 Jul-Aug;99-100:111638. doi: 10.1016/j.nut.2022.111638.

- Ohura T, Nakajo T, Okada S, Omura K, Adachi K. Evaluation of effects of nutrition intervention on healing of pressure ulcers and nutritional states (randomized controlled trial). Wound Repair Regen. 2011 May-Jun;19(3):330-6. doi: 10.1111/j.1524-475X.2011.00691.x.

- Bonetti L, Terzoni S, Lusignani M, Negri M, Froldi M, Destrebecq A. Prevalence of malnutrition among older people in medical and surgical wards in hospital and quality of nutritional care: A multicenter, cross-sectional study. J Clin Nurs. 2017 Dec;26(23-24):5082-5092. doi: 10.1111/jocn.14051.

- **Study type not relevant (n=4)**

- Shafrin J, Wang S, Kerr KW. Wound-Specific Oral Nutritional Supplementation Can Reduce the Economic Burden of Pressure Injuries for Nursing Homes: Results from an Economic Model. Journal of Long-Term Care. January 2023:166-177. doi:10.31389/jltc.173

- Taylor C. Nutrition and pressure ulcers: putting evidence into practice. Journal of Community Nursing. 2016;30(4):38-44. Available on: https://search.ebscohost.com/login.aspx? direct=true&db=rzh&AN=117335424&lang=it&site=ehost-live.

- Wong A, Lai P, Chong HH, Lien CTC, Graves N. The Clinical and Cost-Effectiveness of an Individualized Nutritional CAre (INCA) Bundle versus Standard Care for Adults with Pressure Injuries Receiving Home Nursing Services: A Protocol for a Cluster Randomized and Pragmatic Clinical Trial with an Economic Evaluation. Nutrients. 2024;16(2):299. doi:10.3390/nu16020299

- Chaboyer W, Bucknall T, Webster J, et al. INTroducing A Care bundle To prevent pressure injury (INTACT) in at-risk patients: A protocol for a cluster randomised trial. International Journal of Nursing Studies. 2015;52(11):1659-1668. doi:10.1016/j.ijnurstu.2015.04.018

- **Population not relevant (n=1)**

- Zanini M, Bagnasco A, Catania G, et al. A Dedicated Nutritional Care Program (NUTRICARE) to reduce malnutrition in institutionalised dysphagic older people: A quasi-experimental study. Journal of Clinical Nursing (John Wiley & Sons, Inc). 2017;26(23-24):4446-4455. doi:10.1111/jocn.13774

Paper not selected Other Source (n=1)

- Barateau M, Corompt A, Soulan J, Bourdel-Marchasson I. Multicenter nursing study evaluating the importance of nutritional support in the prevention of bedsores in the elderly at risk. *Rech Soins Infirm.* **1998** Dec;(55):42-9. French.
